# Under-gel Endoscopic Mucosal Resection without Injection: A Novel Endoscopic Treatment Method for Superficial Nonampullary Duodenal Epithelial Tumors

**DOI:** 10.31662/jmaj.2021-0078

**Published:** 2021-09-13

**Authors:** Haruka Amino, Takeshi Yamashina, Hiroyuki Marusawa

**Affiliations:** 1 Department of Gastroenterology and Hepatology, Osaka Red Cross Hospital, Osaka, Japan; 2 Gastroenterology and Hepatology, Kansai Medical University Medical Center

**Keywords:** superficial nonampullary duodenal epithelial tumors, endoscopic mucosal resection, underwater endoscopic mucosal resection without injection, gel immersion

## Introduction

There are no clear criteria for selecting the endoscopic treatment strategy for superficial nonampullary duodenal epithelial tumors (SNADETs). Underwater endoscopic mucosal resection without injection (UEMR) has been attracting attention as a more effective alternative to conventional endoscopic mucosal resection (EMR) for SNADETs ^(1), (2), (3)^. However, water generally flows easily, and we sometimes have to perform EMR even in places where it is hard for water to accumulate. A new gel product (VISCOCLEAR^Ⓡ^, Otsuka Pharmaceuticals Factory, Tokushima, Japan) for endoscopic procedures and treatments was recently launched in Japan, but there have been no reports of endoscopic resection using gel immersion. Thus, we report a novel endoscopic resection method for SNADETs under gel immersion instead of water.

## Case Presentation

### Patients and methods

This study included six consecutive patients with SNADET who were treated from November 2020 to January 2021 at Osaka Red Cross Hospital. The Ethics Committee of Osaka Red Cross Hospital approved the study protocol (No. J-0188). All patients received an explanation of the study and provided informed consent. All lesions were located in areas where water flowed relatively easily and was rather difficult to stay in the lumen. Patients with SNADETs <20 mm were enrolled in the study, following a previous report evaluating the efficacy of UEMR ^(4)^. The under-gel EMR procedures were performed by two endoscopists (A and B). The number of experiences of duodenal endoscopic treatment before this study for endoscopist A (Certification from Japanese Gastroenterological Endoscopy Society) was EMR ≥ 50, and UEMR ≥ 20, and for endoscopist B (Nonexpert) was EMR = 3, and UEMR = 1. The main outcomes of this study were the en bloc resection rate, procedure time, and complications. En bloc resection was defined as endoscopically assessed removal of the lesion in one piece. R0 resection was defined as en bloc resection with histologically confirmed negative horizontal and vertical resection margins. The procedure time was measured from the start of immersion in gel from the endoscope until complete removal of the lesion.

### Under-gel EMR procedure

**Figure 1.**
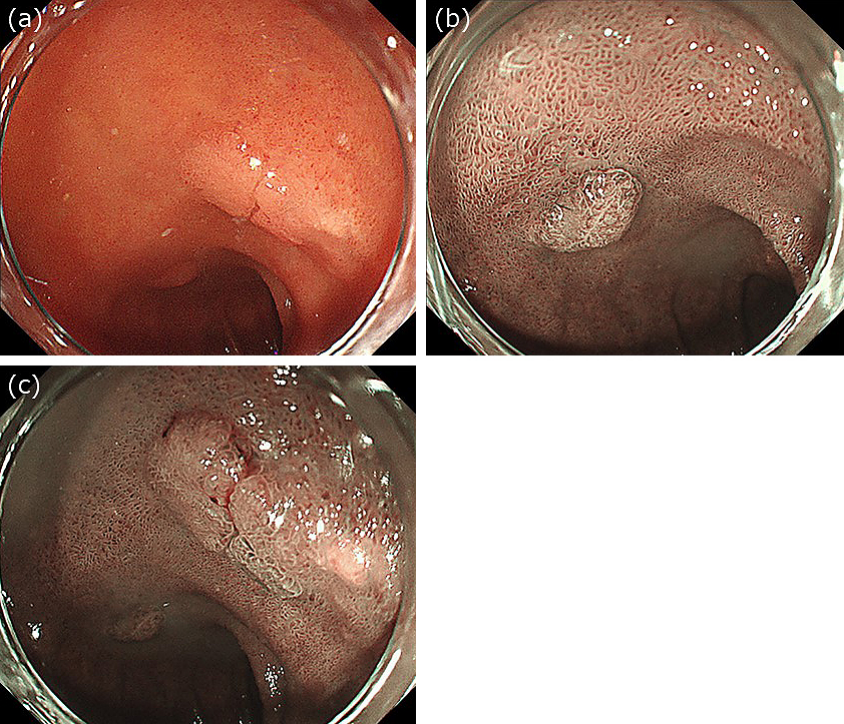
(a) Endoscopic image showing two adenocarcinomas in the duodenum. One lesion is on the superior duodenal angle (SDA), and the other is on the posterior wall of the duodenal bulb. (b) Narrow-band imaging view of the tumor on the SDA. (c) Narrow-band imaging view of the tumor on the duodenal bulb.

**Figure 2.**
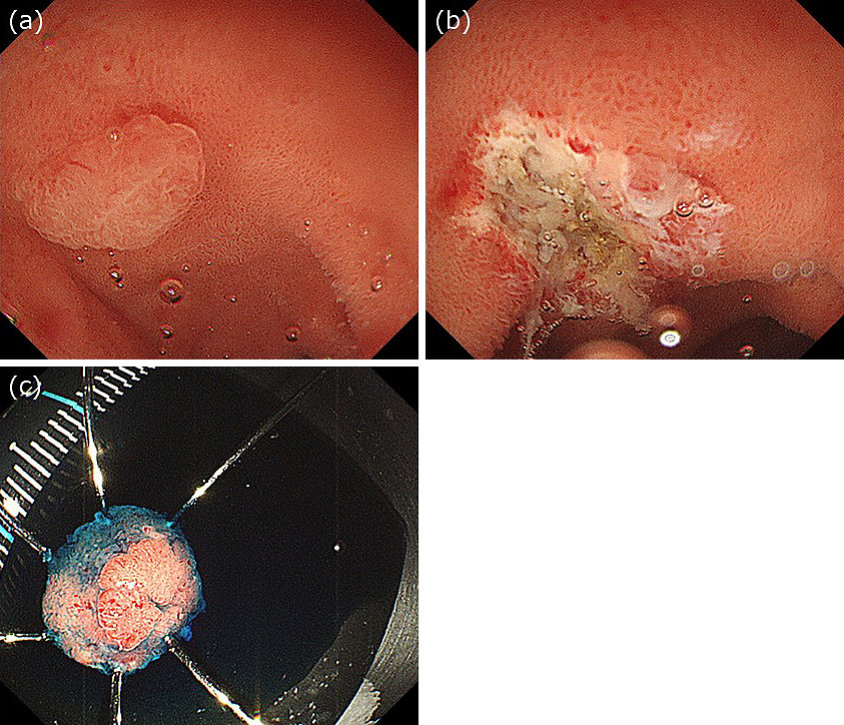
(a) Gel was injected into the duodenal lumen through the accessory channel, and the tumor was immersed. (b) The tumor was completely removed. (c) The resected specimen.

The procedure was almost the same as UEMR. All procedures were conducted using the GIF-Q260J endoscope (Olympus Medical Systems, Tokyo, Japan). Anticholinergic drugs were injected intravenously before the procedure. All patients underwent this procedure in the left lateral position. We deflated the lumen air and injected gel (VISCOCLEAR^Ⓡ^, Otsuka Pharmaceuticals Factory, Tokushima, Japan) instead of water through the accessory channel of the endoscope to fill the lumen. We continued to inject the gel until the lesion was completely submerged (Figure 1a and 1b). The accessory channel was attached to a dedicated part that has two lumens, one for the snare and the other for the gel injection. This way, we can easily add more gel during snaring. After the lesion was fully immersed in the gel, we performed hot snare polypectomy without submucosal injection using a bipolar snare (DRAGONARE™ 26 mm; Xemex, Tokyo, Japan) (Figure 2a and 2b). A high-frequency electrical generator (VIO^Ⓡ^ 300 D; Erbe Elektromedizin, Tübingen, Germany) was used with the following settings: auto-cut mode, effect 3, 30 W; forced coagulation mode, effect 1, 15 W. After the resection was completed, the gel was suctioned as much as possible. The mucosal defect was then closed with endoscopic clips.

### Video case

Two 10 mm duodenal adenocarcinomas were detected in an 80-year-old man: one was located on the posterior wall of the duodenal bulb, and the other was located on the superior duodenal angle (Figure 1). Because it was difficult to perform UEMR in these regions, we injected gel through the accessory channel of the endoscope to fill the lumen (Figure 2a). After the lesion was fully immersed in 100 ml of gel, we performed hot snare polypectomy without submucosal injection using a bipolar snare and a high-frequency electrical generator. We successfully performed en bloc resection with no intra- or post-procedural complications (Figure 2b and 2c). Both lesions were pathologically diagnosed as well-differentiated adenocarcinomas with negative margins (Supplement Material 1).

## Results

Six consecutive patients with SNADET underwent under-gel EMR at our hospital during the study period. Table 1 presents the baseline characteristics of all patients. There were four men and two women, with a median (range) age of 69 (30-80) years. All tumors were located in the duodenum; three were proximal to the duodenal papilla, whereas three were distal to it. The median tumor size was 11 mm (range, 5-15 mm). The outcomes of under-gel EMR are shown in Table 2. In all cases, a total of 100 ml of gel was injected in each. The rate of en bloc resection was 100% (6/6); five cases were R0 resections, whereas one case had unclear horizontal margins. The median procedure time was 6 min (range, 5-10 min). The mucosal defect was closed with endoscopic clips in all cases. The tumors were diagnosed as adenomas in two cases, and as well-differentiated adenocarcinomas in four cases. There was no intraprocedural perforation, delayed perforation, or delayed bleeding.

**Table 1.** Patient Characteristics.

Case	Age (years)	Sex	Tumor location	Tumor location relative to the duodenal papilla	Tumor size (mm)	Macroscopic type
1	67	Female	Second portion	Distal	10	IIc
2	58	Male	Second portion	Distal	10	IIa
3	80	Male	Second portion	Proximal	12	IIa
4	80	Male	Bulbs	Proximal	18	I
5	67	Male	Second portion	Distal	15	IIa
6	71	Female	Second portion	Proximal	7	IIc

**Table 2.** Outcomes of Under-gel Endoscopic Mucosal Resection without Injection.

Case	Endoscopist	En bloc resection	R0 resection	Procedure time (min)	Gel injection volume (ml)	Prophylactic clipping	Histological type	Lympho- vascular involvement	Horizontal/ vertical margins
1	B	En bloc	R0	10	100	7	Adenoma	Negative	HM-, VM-
2	A	En bloc	RX	7	100	11	Adenoma	Negative	HMX, VM-
3	A	En bloc	R0	5	100	4	Intramucosal cancer	Negative	HM-, VM-
4	A	En bloc	R0	5	100	6	Intramucosal cancer	Negative	HM-, VM-
5	A	En bloc	R0	9	100	4	Intramucosal cancer	Negative	HM-, VM-
6	A	En bloc	R0	5	100	4	Intramucosal cancer	Negative	HM-, VM-

## Discussion

Gel immersion endoscopy was first reported as a novel treatment for gastrointestinal bleeding ^(5)^. Although water quickly mixes with the bleeding, which makes it difficult to secure the visual field, injecting a gel-like product instead of water through the attached channel of the endoscope creates a transparent space in front of the endoscope to ensure a good field of view. This makes it easier to identify the source of bleeding and achieve endoscopic hemostasis ^(5), (6), (7)^, even during endoscopic submucosal dissection ^(8)^.

There are no previous reports of EMR for SNADET using the gel immersion method. Although conventional EMR has been commonly used for the treatment of SNADET, in recent years, UEMR has been used as a more effective method because submucosal injection can make snaring more difficult. However, it is difficult to perform UEMR in areas where there is difficulty in attaining water immersion. We think that under-gel EMR may be useful for SNADETs in such areas where it is difficult to perform UEMR. In this study, under-gel EMR achieved a good endoscopic treatment outcome for SNADETs, with a 100% en bloc resection rate and 83.3% R0 resection rate, without any complications. Previous UEMR studies have reported an en bloc resection rate for SNADETs of 73.9%-91.4% and an R0 resection rate of less than 80% ^(2), (3)^. Although the number of cases in the present study was small, both the en bloc resection rate and R0 resection rate were higher than those reported in previous UEMR studies. Additionally, SNADETs were located in the second portion of the duodenum in most of the cases in this study, suggesting that under-gel EMR may be effective in this location. Furthermore, an inexperienced endoscopist was able to perform under-gel EMR easily in one case, suggesting that under-gel EMR has the potential to be used widely in daily clinical practice.

Of course, it would also be possible to immerse the lesion by repeating the processes of air deflation and injection of large amounts of water several times. However, this is time-consuming and may induce peristalsis and make resection more difficult. Previous UEMR studies have reported a median procedure time of 5.9-99.1 min ^(2), (3)^; hence, compared with that using water, the procedure time for under-gel EMR in our study was relatively short because the moderate viscosity of gel makes it easier for the gel to remain in the lumen. Deflating as much as possible will make the lumen smaller and allow the gel to remain in the lumen more effectively.

We conclude that under-gel EMR had a good treatment outcome for SNADETs. This method may be a useful treatment for SNADETs, especially in areas where water immersion is difficult. A multicenter trial is required to confirm the validity of the present results.

## Article Information

### Conflicts of Interest

None

### Acknowledgement

We would like to thank members of the endoscopy room of Osaka Red Cross Hospital for their support.

### Author Contributions

***Conception and design***: Haruka Amino, Takeshi Yamashina, and Hiroyuki Marusawa.

***Analysis and interpretation of the data***: Haruka Amino, Takeshi Yamashina, and Hiroyuki Marusawa.

***Drafting of the article***: Haruka Amino, Takeshi Yamashina, and Hiroyuki Marusawa.

***Final approval of the article***: Haruka Amino, Takeshi Yamashina, and Hiroyuki Marusawa.

***Guarantor of the article***: Takeshi Yamashina.

### Approval by Institutional Review Board (IRB)

J-0188, The Ethics Committee of Osaka Red Cross Hospital

## Supplement

Supplement Material 1 Two adenocarcinomas in the duodenum were successfully resected by endoscopic mucosal resection under gel.
